# Legislative and judicial responses to public health protection under eco-environmental damage relief in China

**DOI:** 10.3389/fpubh.2023.1197636

**Published:** 2023-07-06

**Authors:** Huaqi Li, Bei Liu

**Affiliations:** ^1^School of Law, Hohai University, Nanjing, China; ^2^School of Management, Nanjing University of Posts and Telecommunications, Nanjing, China

**Keywords:** ecological environmental damage, public health, ecological environmental restoration, public law norms, judicial implementation

## Abstract

**Background:**

The group-type health damage caused by eco-environmental damage has been stated in the Environmental Protection Law and other laws in China. The first-ever Chinese Civil Code, which took effect in 2021, has explicitly defined eco-environmental damage relief and imposed affirmative duties on those who polluted the environment or destroyed the ecology. This study aims to describe the status quo of public health protection in eco-environmental damage relief and explore its progress and limits in protecting public health.

**Methods:**

By reviewing China’s legislation on relief of eco-environmental damage and observing the implementation of these laws in judicial practice. All judicial cases of eco-environmental damage published by Chinese courts from January 2021 to May 2023 were selected and examined. From the perspective of the comparison of laws, the measurement of interests and the execution of cases, we discussed the issues of China’s legislative and judicial responses to public health.

**Results:**

The relief system of eco-environmental damage in China has been formed initially, but there are still some deficiencies: In the application of law for relief of public health, there are many choices of means, resulting in the choice conflict of law application; the public health damage in the eco-environmental damage case has been ignored by courts, and it has not been a dominant consideration element; the objects of the execution of the cases are directed to the pure eco-environmental restoration, and the restoration plan does not cover public health protection measures, which does not have a preventive effect on public health. The root cause of problems is that the relief of eco-environmental damage in China follows the logic of traditional private law.

**Conclusion:**

The issues mentioned above merit consideration in China’s future law revisions and judicial practice. Based on the dual nature of public and private law in environmental health, it is necessary to adjust the provisions of responsibility for eco-environmental restoration from the framework of public law, including the scope and mechanism, and then further suggestion includes the legal subject, the benefit element and the use of funds.

## 1. Introduction

The increasingly prominent environmental issues, especially the frequent occurrence of high-level pollution of the environment and serious ecological damage events, have made ecological environmental damage a major social issue that must be addressed urgently. In recent years, the Guangxi Longjiang cadmium pollution incident,^1^ blood lead poisoning of children in Hengyang (Hunan Province),^2^ and the Xiangshui “3·21” especially serious explosion accident in Jiangsu Province^3^ have all reflected the gravity of current environmental health problems. In 2016, the “Outline of the Healthy China 2030 Plan” formulated by the CPC Central Committee and the State Council pointed out that health is an inevitable requirement for promoting all-round human development and a basic condition for economic and social development. The report of the 20th National Congress of the CPC further calls for “promoting the construction of healthy China” and “giving priority to the strategic position of development to protect people’s health” in the section on “Improving people’s well-being and improving people’s quality of life,” and stresses the construction of beautiful China in the section on “promoting green development and promoting harmonious coexistence between man and nature.”

The World Health Organization Act states: “Health is not merely the absence of disease or infirmity, but a state of complete physical, mental and social well-being” (1). This means that the meaning of health should include both physical and mental health and the healthy state of the social environment. The correlation between public health and ecological environmental damage is manifested in two aspects: First, when environmental pollution affects human health, it usually means that individuals suffer direct and actual harm to their personal and property rights and interests, and this is personal damage, which can be remedied through traditional tort damage relief channels. Second, amidst the current prominent environmental problems, including large-scale environmental pollution, soil, air, water, and other elements are poisoned and the ecosystem deteriorates, resulting in the health of people living in the polluted or damaged areas being harmed. This kind of damage is latent to a certain extent, and tends to affect public health after long-term accumulation, resulting in group-type health damage (2). An operational framework based on a transdisciplinary definition of Socio-Ecological System Health (SESH) has been proposed that explicitly links health and ecosystem management with the resilience of the Socio-Ecological System (SES) (3). It can be seen that this kind of damage is no longer a simple case of civil tort, but also involves social public security issues. From the level of normative documents, this kind of damage can be summarized as environmental health damage, which specifically refers to the disease-type health damage and hidden danger-type health damage caused by environmental pollution (biological, chemical, and physical) to public and individual health.^4^ Based on this, this paper will focus on the protection of public health damage under the relief of ecological environmental damage.

On May 28, 2020, the third session of the 13th National People’s Congress passed the Civil Code of the People’s Republic of China (Civil Code), which added the provisions on ecological environmental damage liability (Articles 1,234 and 1,235) after the provisions on environmental tort liability in the tort liability section, officially incorporating eco-environmental damage liability into the civil liability system and becoming a substantive norm for ecological environmental damage relief. As the “Green Civil Code” comes into effect, the application of the provisions on eco-environmental restoration responsibility as a special clause that distinguishes it from other tort liability provisions has become an important topic. Some scholars have discussed this issue from four perspectives: first, starting with the legal issues of eco-environmental damage compensation provisions, analyzing the basis of the civil law ownership system and the principle of liability for the eco-environmental damage compensation system (4, 5); second, based on the binary distinction between ecological environmental damage responsibility and private tort liability, exploring the attribution principle, the form of responsibility, and the application of the connection with other norms of the ecological environmental damage responsibility provisions (6–8); third, clarifying the substantive status of the eco-environmental restoration responsibility provisions in the Civil Code, sorting out the priority relationship between eco-environmental damage compensation litigation and environmental civil public interest litigation (9, 10); and fourth, focusing on the legal application issues between eco-environmental restoration by proxy in the Civil Code and environmental administrative performance (11). It can be seen that China has initially formed a relief system for ecological environmental damage with civil law as the core. Articles 1,234 and 1,235 of the Civil Code can provide relief for such group health damage by means of restoration or compensation for eco-environmental damage (EED).

## 2. Literature review

The research on remedies for EED should first clarify the ecological environment category. In the field of law, “ecological environment” is used as an object, which refers to the objectively existing natural environment, including all kinds of material entities and natural space in nature, especially the living system or ecosystem. Meanwhile, it emphasizes the existence of all kinds of substances as an interconnected whole, which not only points to the environment itself but it also refers to the “environment” as the living condition of human beings, namely the material environment centered on human beings (12). Ecological damage is usually measured in terms of the cost of the restoration action required to provide benefits sufficient to offset the loss of ecological resources or services (13). Therefore, eco-environmental restoration refers to taking measures to restore the damaged ecological environment to its original or expected state, but its connotation needs to be defined at a more specific level. In terms of restoration objectives, it is generally believed that the ecological environment should be restored to its original state. However, given the dynamic evolution characteristics of natural systems, it is not easy to determine what the original state is and whether it is worthy of being the target of restoration is also questionable. Therefore, the expected state, namely the ecological environment state determined by certain procedures, may be more suitable as the target of restoration, and be concretizde as “repair degree” (14). In terms of restoration content, it is necessary to define this more specifically according to its objectives, and clarify the methods, contents, indicators, and even the technical plans for environmental pollution cleaning and ecosystem repair. Cleaning up pollution is usually a necessary form of remediation, and further such measures are determined by taking into account species, space, and ecological sustainability and renewal. In December 2022, the Ministry of the Ecology and Environment issued the Technical Guidelines for Assessing the Effectiveness of Ecological Protection and Restoration (Trial; HJ1272-2022). It is made clear that the evaluation indicators of ecological and environmental restoration effectiveness include important ecosystem area, ecological connectivity, and natural shoreline retention rate, vegetation coverage, and environmental quality, biodiversity, leading ecological functions, human stress, public satisfaction, and characteristic indicators. In practice, restoration includes a set of activities and goals related to reversing ecosystem degradation and strengthening ecosystems as a whole (15). As Reports by the Society for Ecological Restoration (SER), these series of activities are interconnected. However, different types of activities have different baseline requirements or objective goals (16). When the responsible subject is unable to actually take measures, the legal subject can request the responsible subject to pay expenses according to the cost of the corresponding repair measures through legal channels, namely compensation.

## 3. Development of legislation for relief of eco-environmental damage in China

In China, the National People’s Congress and its Standing Committee have the power to make national laws and may authorize the executive branch to develop regulations. Based on the Constitution, the following national laws and department regulations were passed to address environmental pollution or ecological destruction (as summarized in Table 1).

**Table 1:** Legislation about environmental restoration.

Title	Issuing authority	Enactment date	Amendment date	Provisions (Keywords)
The civil code	National people’s congress	2020-5-28	—	Article 1,234: violation of state regulations; eco-environmental damage; the organ prescribed by the state; the organization prescribed by law; the liability for restoration; substitute repair; and expenses. Article 1,235: losses; expenses.
Environmental protection law	The standing committee of the national people’s congress	1989-12-26	2014-4-24	Article 58: public interests; social organizations; register according to law; engaging exclusively in public welfare activities of environmental protection for at least five consecutive years and having no illegal record.
Civil procedure law	National people’s congress	1991-4-9	2017-6-27	Article 58: public interest; polluting the environment; the organs; organizations prescribed by law; and institute proceedings before the People’s Court.
Administrative enforcement law	National people’s congress	2011-6-30	_	Article 50: administrative organ; make decision; perform obligation; environmental pollution; damage natural resources; perform on its behalf; and entrust a third party.
Regulations on the administration of compensation for eco-environmental damage	Ministry of ecology and environment, ministry of natural resources, SPC, etc.	2022-4-26	_	Article 9: the designated department; institution; obligator; restoration for EED; the baseline level before the damage; the level at which risks to the ecological environment are acceptable; and compensation.

### 3.1. Environmental protection law (2014)

The Environmental Protection Law (EPL) is the first law in China to focus on compensation for damage to the ecological environment. Article 58 specifically stipulates that social organizations that meet the legal conditions can file environmental civil public interest lawsuits to safeguard the public interests in this regard. Environmental public interest litigation (EPIL) by social organizations has become the beginning of China’s relief system for EED. However, due to the small length of this article, it only stipulates that the subject qualification of legal social organizations, and does not make clear provisions on qualification conditions, damage degree, litigation claims, liability forms, etc. In addition, prosecutors have brought environmental public interest lawsuits in practice, but the article does not mention these prosecutors or other subjects. This provision means that the practice of EPIL is not smooth. However, most environmental public interest lawsuits filed by social organizations at this stage have been rejected by the court as not meeting the legal conditions (17).

### 3.2. Civil procedure law (2017)

In December 2014, the Supreme People’s Court (SPC) issued the Interpretation on Some Issues concerning the Application of Law to the Trial of Environmental Civil Public Interest Litigation Cases, which made a series of provisions on the subject hierarchy, as well as the scope procedure, and claim of litigation, further specifying the legal subjects to bring EPIL, ushering in an era of EPIL in China. In 2017, the Civil Procedure Law was amended to add provisions on civil public interest litigation in the environment field. Article 58 clearly grants the corresponding EPIL rights to the organizations and organs prescribed by law, which mainly refer to procuratorial organs. In 2020, the SPC amended the above judicial interpretation, made detailed provisions on the conditions of legal social organizations, and added that procuratorial organs can start litigation proceedings without social organizations filing lawsuits. In addition, Article 26 specifically adds that departments responsible for environmental and resource protection supervision and management should perform their regulatory duties in accordance with the law so as to restore the environment, and these subjects shall not bring a lawsuit.

### 3.3. The Civil Code (2020)

The first Civil Code in China, which was enacted on May 28, 2020 and took effect on January 1, 2021, is aimed at clarifying, integrating, and amending existing rules in the field of Chinese private law. Among them, the seventh chapter of the tort liability series stipulates the liability for environmental pollution and ecological damage. Article 1,229–1,233 of Civil Code stipulates the specific applicable circumstances of environmental tort (tort liability, causality, damage situation, punitive compensation, and third-party fault); and Article 1,234–1,235 of Civil Code stipulates the liability for the scope of restoration and compensation of the damage to the ecological environment caused by the actor. The questions are: Which entities can be held responsible for ecological and environmental restoration? How can the responsibility for eco-environmental restoration be identified? What is the scope of the responsibility for ecological and environmental restoration? and How can the liability for compensation be determined when the liability cannot be borne?

### 3.4. Administrative enforcement law (2011)

According to Article 50 of Administrative Enforcement Law, environmental displacement of fulfillment is the administrative power of the state organ in the public law, and it is a way to remedy the damage to the ecological environment. In addition, various separate laws, such as the Law on the Prevention and Control of Water Pollution, the Law on the Prevention and Control of Environmental Pollution by Solid Waste, the Law on the Prevention and Control of Soil Pollution, the Water Law, and the Forest Law and so on, also clearly stipulate provisions on displacement of fulfillment, and the ways of restoration vary according to different environmental factors. The essence of the eco-environmental restoration system stipulated in the Civil Code is to introduce legal organizations as rights subjects in private law, add public subjects, simplify the implementation procedure, and realize the protection of environmental public interests. However, the relationship between the Civil Code’s ecological environmental restoration responsibility clause and the environmental displacement of fulfillment clause in public law has not been clarified.

### 3.5. Regulations on the administration of compensation for eco-environmental damage (2022)

In response to the problem of insufficient administrative punishment, a 2-year-period pilot program of eco-environmental damage compensation litigation (EEDCL) was launched in seven designated provinces in December 2015, 5 months after the start of the procuratorate-initiated PIL pilot program. As authorized by the State Council, the provincial governments of pilot areas have been granted standing in EEDCL and can entrust the local environmental authorities to file EED lawsuits against ecological abusers (18). In May 2019, the SPC issued the Provisions on the Trial of Eco-Environmental Damages Litigation Cases (Trial) to solve the issues in judicial practice and to make procedural arrangements. In 2022, 14 departments, including the Ministry of Ecology and Environment, the SPC, the Supreme People’s Procuratorate, and the Ministry of Natural Resources, issued the Regulations on the Administration of Compensation for Eco-Environmental Damage (“Management Regulations”). Arguably, the EEDCL is one kind of EPIL where qualified government agencies are the plaintiffs with specific claims, i.e., financial compensation against severe ecological infringements, which sets a mandatory pre-trial procedure, namely that government agencies are only allowed to initiate the EEDCL if they fail to reach a compensation agreement with an ecological destroyer by negotiation. The problem is, it shares the same purpose as environmental civil public interest litigation, i.e., the protection of environmental public interest similarly to other litigation procedures. The Civil Code tries to integrate the two types of litigation together, but it still lacks the corresponding connection of litigation procedures.

## 4. Reflection: the protection of public health under eco-environmental damage relief in China

### 4.1. The dilemma of law application choice

According to Article 1,234 of the Civil Code of China, the subjects of the eco-environmental restoration liability clause involve two categories: one is the liability subject who directly causes ecological environment damage, namely “the subject who violates national regulations and causes ecological environment damage”; the other is the subject who exercises the right of eco-environmental restoration, namely “the organ prescribed by the state or the organization prescribed by law.” Due to the complexity of eco-environmental restoration, it often faces many practical operational problems when the illegal subject implements eco-environmental restoration measures on its own, especially when the specific restoration obligation of relevant subjects has not been clearly defined by environmental legal norms. Therefore, eco-environmental restoration is usually carried out by statutory subjects through representative restoration.

Before the promulgation of the Civil Code, Article 50 of Administrative Enforcement Law clearly stipulated that the administrative authority might require the party concerned to fulfill obligations such as eliminating obstacles and restoring the original state, which can be regarded as incorporating environmental protection and restoration into the system of substitute performance. Specifically, the implementation of environmental administrative performance should include the removal of pollution, environmental remediation, and eco-environment restoration (19). Among these, pollution removal is the precursor to environmental remediation, which refers to the process of blocking, controlling, removing, transferring, fixing, and disposing of pollutants in the ecological environment. Environmental remediation is a measure taken to further reduce the concentration of pollutants in the environment after the completion of pollution removal. The goal is to reduce the human health risk or ecological risk caused by environmental pollution to an acceptable level. Eco-environment restoration goes further. It is the process of restoring the damaged ecological environment and its service functions to the baseline and compensating for the damage during that period, including both environmental and ecological service function restoration, which has the same meaning as “ecological environment restoration” stipulated in the Civil Code.

**Figure 1:**
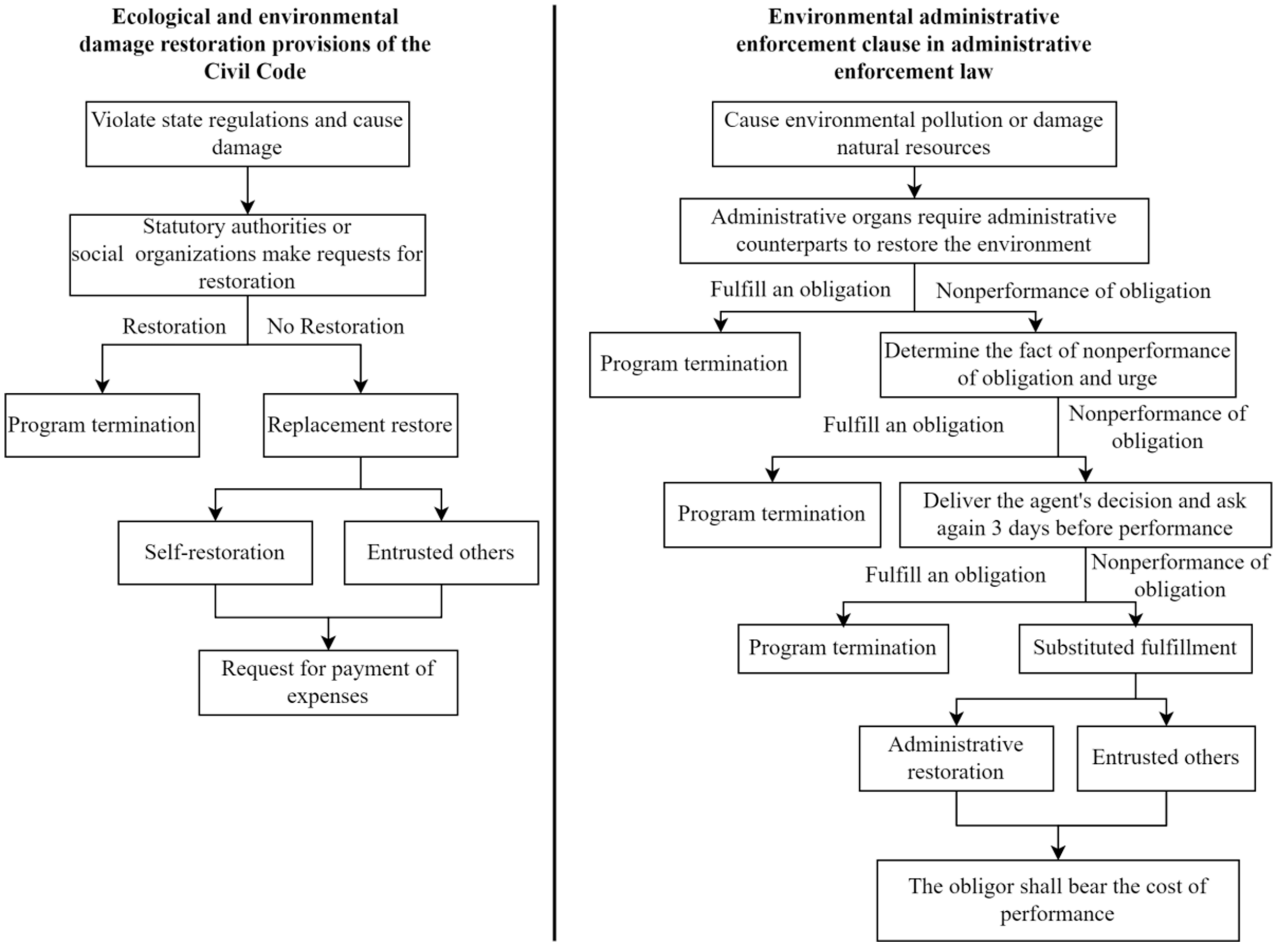
Contrast between eco-environmental restoration and administrative agent performance.

As for the difference between ecological environment substitute repair and ecological environment administrative substitute performance, the former is mainly implemented through judicial power (such as environmental civil public interest litigation/ecological environment damage compensation litigation) and is expressed as “restoring the original state” and “compensating for losses” in the litigation request. The latter requires the administrative authority to make an administrative decision requiring the administrative counterpart to perform certain actions, and to issue multiple notices when said counterpart fails to perform the obligation, and if they still fail to do so, the administrative authority shall implement substitute performance (self-perform or commission others to perform) and they shall bear the performance costs (20). It can be seen that in terms of eco-environmental restoration, China has at least has ways of achieving environmental administrative substitute performance, EPIL, and EEDCL (Figure 1).

However, when the preceding two clauses appear concurrently, the administrative authority, as the subject capable of simultaneously exercising the environmental administrative agency and the eco-environmental restoration, will still face great uncertainty in choosing to request restoration based on the provisions of the eco-environmental restoration liability clause of the Civil Code. At the same time, based on the right of restoration in the Civil Code provisions, when eco-environmental restoration is necessary, the claimed right of replacement repair can be chosen by the legal subject. Once involved in the restoration process, the legal subjects will be subject to public supervision in terms of repair plans, implementation, effects, etc. In contrast, China has clear legal provisions and guarantee mechanisms for the environmental administrative agency’s delegated performance, which can not only remedy existing ecological environmental damage, but also prevent it from occurring. If the administrative execution power can achieve the purpose of restoration, the administrative organ will definitely prioritize the performance of legal duties, exercise delegated performance power, and abandon the right to request delegated repairs. Therefore, the ecological environment repair liability clause will become a “mere scrap of paper.”

### 4.2. Challenge in identifying public health interests

**Figure 2:**
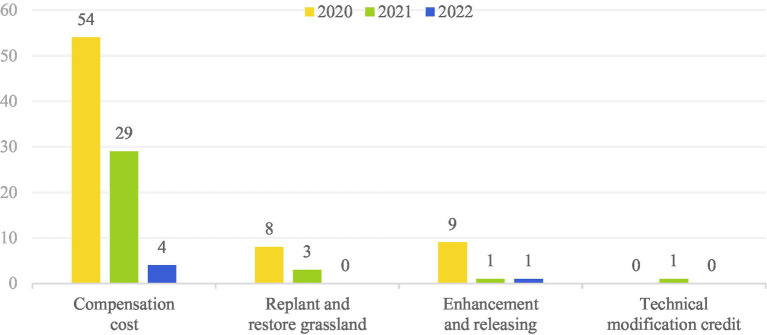
Innovative form of judgment. Source: China Judgments Online.

Since the implementation of the Civil Code, there has been a controversy about the identification of “ecological environmental damage” in theory and practice circles. On December 23, 2018, the Constitution and Law Committee of the National People’s Congress (NPC) issued a draft on Tort Liability of the Civil Code. The Report on the Modification clearly distinguishes “personal and property damage of civil subjects” from “damage to the ecological environment itself, “thus presuming that the damage to the ecological environment refers to adverse changes in the physical, chemical, and biological characteristics of the ecological environment itself and the destruction or harming of the ability to provide ecosystem services.” According to Article 1,235 of the Civil Code, the loss and expense that the infringer should compensate for the environmental damage include: the loss caused by the damage to the ecological environment and the loss of the service function during the restoration period; losses caused by permanent damage to ecological and environmental functions; expenses for investigation, appraisal and assessment of EED; expenses for cleaning up pollution and restoring the ecological environment; and reasonable expenses incurred to prevent the occurrence and expansion of damage. Therefore, the provision on eco-environmental restoration responsibility is regarded as an expansion of the scope of civil law incorporating ecological environmental damage into the category of civil law damage (21). As the largest developing country in the world, China is not only facing serious environmental pollution, but is also one of the developing countries with the most experience in implementing environmental regulation policies (22). Article 4 of Management Regulations stipulates that the scope of application of EEDCL does not include situations involving personal injury, individual and collective property loss and Marine EED, and once again emphasizes the relief of EED itself. Similarly, Article 58 of the Civil Procedure Law points the scope of application of environmental civil public interest litigation at the behaviors that damage social public interests such as environmental pollution, but does not cover the ecological environmental damage situation with the loss of private rights and interests. Based on the above-mentioned legal provisions, judicial practice presents the exploration of the restoration or compensation of the pure ecological environment. For example, the forms of “replenishing planting and restoring green,” “enhancement and releasing,” and “technical modification deduction” appear to be alternative repair methods. Among them, 160 million EPIL cases in Taizhou adopted the method of “technological modification deduction,” which brought into question the responsibility for eco-environmental restoration.^5^ Through the search for eco-environmental restoration and compensation cases in the past 3 years, it can be seen that under the ecological environment damage relief system of civil law, the compensation for environmental pollution and public health damage has undoubtedly become a “vacuum zone” for relief. First, the public health damage of environmental pollution involves the health rights and interests of an unspecified majority of people, has obvious public interest attributes, and exceeds the private interest category of individual health damage, so it is difficult to apply environmental private interest litigation to remedy it. Second, the relief approach of ecological environmental damage is aimed at maintaining the public interest in the environment. The focus of relief lies in the damage of the ecological environment itself, and the damage of public health is not given any attention. The damage to public health caused by environmental pollution or ecological destruction appears to be marginal (Figure 2).

Based on the equality and autonomy of the civil tort relief system, the damage relief should be limited to the harm caused to the civil subject’s rights and interests. Some scholars have pointed out that environmental public interest is not a civil right and that direct relief of ecological environmental damage is incompatible with the private nature of civil law (23). Once the EED is included in the civil law, the eco-environmental restoration responsibility clause should have the dual purpose of protecting interests of the ecological environment and public health. The liability clause of eco-environmental restoration emphasizes the protection of such interests. Due to the scope of the private law system, it should still follow the logic of law application of the private law system, and the harm to be restored should be directly related to the environmental tort damage, that is, it is not to remedy the eco-environmental harm that has nothing to do with the damage of private rights and interests.

### 4.3. Prevention function issue remain unsolved

Because of the hidden and delayed nature of health problems themselves, public health protection is more focused on the prevention of such risks, rather than merely the after-action relief in the face of real damage. This means that the protection of public health should not only be limited to the damage already caused, but also pay attention to the prevention of the risk of environmental pollution to public health, and reduce the threat of harm caused by such pollution or ecological damage so as to avoid the irreversible consequences of damage to health (24).

Based on the relief approach to ecological environmental damage under the framework of civil law, environmental civil public interest litigation and EEDCL (25) are mainly integrated and unified. To be specific, the litigation proceeding carried out with the aid of tort relief rules still focuses on the generation of damage consequences, that is, the EEDL is limited to the specific situation of “serious impact on ecological environment consequences,” and the pollution or damage has a large and wide-ranging impact, and may even affect the surrounding residents. The scope of environmental civil public interest litigation is obviously much broader and can be applied to all public interest damage caused by environmental pollution or ecological harm. At present, only the judicial interpretation of environmental civil public interest litigation clearly mentions that “circumstances with major environmental risks” can initiate litigation. Preventive action of this kind has also appeared in practice. In the Yunnan Green Peacock case,^6^ Friends of Nature argued that the defendants (Xinping Corporation and Research Institute of Kunming Engineering Corporation Limited) had physical and procedural problems with their EIA report for a hydropower project that would inundate the green peacock’s main habitat and likely lead to its extinction. As a typical case of preventive environmental civil public interest litigation, the final decision was to suspend the project. Although the case played an objective role in preventing environmental health risks, the lawsuit did not focus on the health protection of the public and only took species extinction as the point of argument for environmental risks.

Overall, there are fewer judicial cases of environmental risk regulation, because the scientific uncertainty of environmental risk causes obstacles to judicial identification. The most typical is climate change, which has been proven scientifically to be caused by greenhouse gas emissions and is bound to have adverse effects on human health. Studies have shown that “climate change is influencing the rate at which toxic chemicals are released from plastic materials, stockpiles and polluted sites” (26), but the impact of such adverse changes on human health is not immediately apparent currently, so it is difficult to get support for the relief of ecological environmental damage. In other words, the difficulty in preventing public health risks lies in the fact that it is hard to prove the causality of environmental health damage for the damage in the latent period based on medical standards, and it is particularly problematic to provide judicial remedies for such harm caused by environmental issues in practice (27). The purpose of environmental protection is twofold: first, to protect the right to survival with the basic needs of the public as the core; and second, to protect the right to progress with sustainable development as the core. The way to protect public health through the ecological environment is to first ensure that the public does not get sick due to environmental pollution or ecological destruction, and then to enhance their physical fitness to improve their health level. In comparing the operational cost of damage relief and prevention, the latter is the most economical and efficient approach. In the field of environmental management, the change from post-relief to pre-prevention can best meet the public’s health needs.

## 5. Legal interpretation under the logic of the integration of public and private law

### 5.1. China’s logic: adopting public law norms in the Civil Code

The provisions on eco-environmental restoration liability in the Civil Code can be regarded as a public law norm within said Code. From a public law perspective, it serves as the fundamental provision and basic norm of the entire system for remedying ecological damage in the country. This normative understanding requires a preliminary issue to be addressed: how the Civil Code, which is of a private law nature, can break through the basic principle of the division between public and private law to establish the legitimacy of a public law norm.

**Figure 3:**
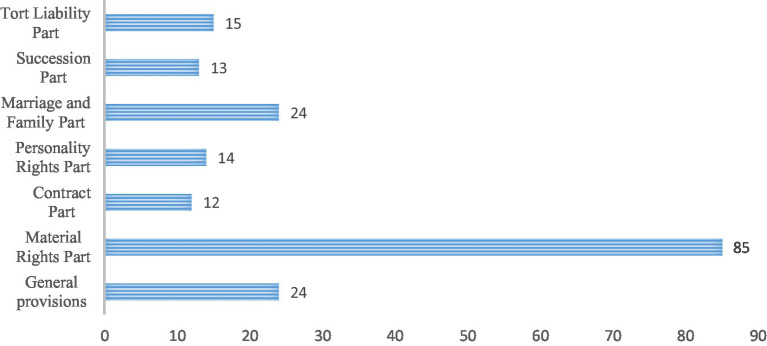
The distribution of the public law norms in the Civil Code.

From the perspective of the development of private law, it seems that public law matters can find a basis within the system of the former, as general legal principles mainly originate from private law (28). However, the emergence of new social law has broken the dominance of private law and formed two paths of public and private law. The law cannot forget its common purpose, and private law must still serve the common good while at the same time responding to individual interests (29). The Civil Code, as a highly systematized product and the crystallization of traditional private law norms, must inevitably be connected and in dialogue with public law norms, and therefore needs to incorporate public law tasks and play, to a certain degree, a public law role, which is essentially the embodiment of “holism and pluralism” (30). In fact, public law norms are not limited to the environmental field in the expression of the Civil Code, but exist in various forms (e.g., public law subjects, acts, qualifications, responsibilities, bases, and interests). As shown in Figure 3, there are corresponding public law norms in each civil Code, including the article of public law in material rights part accounts for the highest proportion. Article 326 of the Civil Code stipulates that the owner of natural resources (state or collective) shall not interfere with the exercise of usufructuary rights, etc., which fully reflects the thought that “the exercise of ownership should be beneficial to the social public interests,” and then determines the rules of public interest expropriation, adjacent relations, and building ownership separation. This kind of public law norm is based on the social public interest in protecting, or intervening to a certain degree in, individual rights and interests.

In terms of normative function, the public law norms in the Civil Code can be divided into two categories: the first is provisions made to protect the realization of private rights, such as the aforementioned public information of registration authorities, which can maintain the consistency between the public status and the true status of rights and maintain market security; the second is the incorporation of external public law norms into the civil law system through normative induction, which not only guarantees the application of civil law rules but also enables the invocation of external public law norms, as in the exercise of natural resource ownership mentioned earlier. The clause on eco-environmental restoration responsibility conforms exactly to these two normative characteristics. On the one hand, the clause can protect ecological and individual environmental rights and interests, that is, it can safeguard certain individual environmental rights while maintaining environmental public welfare, which is helpful in improving the “environmental tort liability system” and forming a complete “environmental damage relief system” covering both public and private interests (31). On the other hand, the clause integrates the long-practiced litigation procedures, but at the same time, judicial practice cannot completely abandon the rules of traditional tort law. Positioning the clause on eco-environmental restoration responsibility as a “public law norm” not only provides a substantive legal basis for “green litigation” but also leaves space for the connection between environmental law and the Civil Code, which is helpful in realizing the transition from the “private law model” of ecological environmental damage relief to the “public law model.” Therefore, the clause on eco-environmental restoration responsibility is a conversion and extension of public law mechanisms into the private law field and should be interpreted and applied as an “expansive clause” according to the operating rules of such mechanisms (32). Based on this position, it is possible to establish an eco-environmental restoration responsibility system within the framework of public nuisance, thereby emphasizing eco-environmental restoration (33).

### 5.2. Discussion on the scope of law application

As a public law norm in the Civil Code, it is necessary to clarify the application scope of public law norms in the private law system to avoid confusion between the public and private law systems caused by the eco-environment restoration liability provisions. Environmental cases in practice can be divided into three categories: disputes over personal rights infringed by environmental pollution or ecological damage, pure EED, and disputes where both eco-environmental harm and personal rights infringement coexist.

The first type of dispute directly manifests as “harm to individuals,” and the interests involved in such disputes still belong to the private interest category and should be governed by environmental tort rules, with special tort rules in the tort liability system providing relief. At the same time, environmental tort litigation can protect environmental interests by means of elimination of obstacles, restoration of the original state, etc. However, the interests involved in this type of dispute are fixed; it cannot achieve the overall protection of the ecological environment system. The second type of pure EED refers to the interest harm suffered solely by the ecological environment, which is par with personal injury and property damage caused by environmental tort (34). This definition takes into account the ecological value of the environment, namely the damage to ecological service functions outside of property damage. When the affected environment involves the environmental interests of an unspecified majority or the state’s ownership of natural resources, the limitations of the remedies under the private law system determine that administrative measures should be given priority for relief and restoration. At this point, the eco-environment restoration responsibility clause should be applied in combination with the specific circumstances. The third type of dispute interest involves both public and private interests，which needs to be further refined. If environmental pollution or ecological damage has an adverse effect on individual interests, the victim can be relieved through private interest litigation. For this part of individual health damage, “adhere to the idea of first identifying private interests and then identifying public interests, and reverse exclude the scope of determining public interests” (35). That is to say, when the damage of private interests is involved, such as the damage of individual right to health, collective ownership of natural resources and private right to use, the private interests damage should be directly excluded from above provisions, which can be divided into “the private interests exclusion in the restoration of EED and the private interests exclusion in the compensation of EED” (36). If the damage to the ecological environment will adversely affect the health of an unspecified majority of people, it should be included in the scope of the implementation of the liability clause for eco-environment restoration. Public health damage is not directly affected by the environment damage like as physical health damage, and is a kind of indirect and delayed group-type health damage, which has negative effects on human health through environmental media. The relief of ecological damage in private law usually has the characteristic of passive defense, and the standard to measure whether the right to health has been violated is the medical standard, that is, the standard of liability for the occurrence of disease. In contrast, the liability for eco-environment restoration clause, viewed from the perspective of public law, can cover damage to public health beyond the traditional right to health. As described in judgment document, “Human beings can only maintain their life by breathing air. If human beings live in an environment with excessive pollutant content in the air for a long time, it will inevitably pose a threat to human health and even endanger their lives.”^7^ In other words, the impact of EED is not only limited to individual citizens, but also includes potential citizens who may suffer from environmental health pollution. In this case, it is necessary to include public health into the scope of relief for EED, showing the relief and prevention function of the eco-environmental restoration responsibility clause on public health damage.

Due to the traditional tort rules only remedy the problem of personal injury and property right, which has clear directivity, so it cannot be extended to cover the scope of public environmental health. Although the eco-environment restoration clause belongs to the private law system, it is still in essence a response to the public affairs problem (Refer to public interest). Besides focusing on the pure EED, it should also pay attention to the public health damage affected by such damage, that is, the potential or widespread health impact of environmental pollution or ecological damage on the surrounding residents through the media.

### 5.3. Integration of law enforcement mechanisms

In the absence of a specialized legal framework for remedying EED, the urgency of ecological restoration necessitates the establishment of basic rules under the Civil Code. Based on the theoretical argument of public law norms, it is feasible to regard the ecological restoration liability provisions as the “fundamental clause and basic norm of the entire public law ecological damage compensation system of the country.”

Based on the positioning of public law norms, the implementation mechanism of the eco-environmental restoration liability provision needs to address a core issue. During the process of compiling the Civil Code, the integration of two types of lawsuits, namely litigation of civil public interest and that of EEDCL, became one of the main objectives of the implementation of the eco-environmental restoration liability provision. Looking at the expression “national organs prescribed by the state” in Article 1,234 of the Civil Code, the subject of the eco-environmental restoration liability system should not be limited to the subjects of the right to sue in the litigation of civil public interest and EEDCL but should also include administrative organs that enjoy statutory environmental administrative performance power. First, the environmental administrative substitute enforcement mechanism conforms to the purpose of eco-environmental restoration. This mechanism emphasizes that administrative organs should clearly order infringers to carry out eco-environmental restoration, urge them to fulfill their restoration obligations if they fail to do so and may perform the restoration on their behalf if they still do not fulfill their obligations. In practice, some administrative organs have been sued by prosecutors for failing to dispose of hazardous waste in a timely manner and believe that the power of performing the restoration on behalf of the infringer is an administrative one of these organs.^8^ Furthermore, the State has a public legal obligation to guarantee public access to a healthy environment, which is a fundamental aspect of the right to health and a prerequisite for the protection of the public’s right to environmental health. Article 1,234 of the Civil Code clearly stipulates that ecological environmental damage behavior should meet the criterion of “violating national regulations.” After such violations occur, state organs and organizations prescribed by law may request infringers to bear the restoration liability within a reasonable period of time. If the infringer fails to carry out the restoration within the time limit, they may do so by themselves or entrust others to do this task, and the cost shall be borne by the infringer. By comparison, administrative organs are authorized to make a series of decisions, such as urging, ordering, and performing the restoration based on their official duties, which is consistent with the content of the eco-environmental restoration liability clause established in the Civil Code and ultimately points to the purpose of eco-environmental restoration. Secondly, the environmental administrative enforcement mechanism is of the same nature as “green litigation.” In the process of performing activities, the actor should strictly comply with national laws, regulations, standards, and other provisions. Once they violate national regulations and cause EED, relief will be provided through the assumption of eco-environmental restoration liability. Currently, the implementation mechanism of the eco-environmental restoration liability clause is mainly manifested in civil litigation procedures led by the judicial authority (EPIL/EEDCL), with the former positioned as “enforcement on behalf of law” (37)，and the latter seen as “public litigation” (9), both of which are in the category of public law liability. Combined with the provisions of Article 50 of the Administrative Compulsion Law, which includes departments for the management of the ecological environment, water, fishery, and other entities, based on the environmental enforcement power, they can not only directly order the actor to restore, but also do this on their behalf, and can also request the restoration of EED. Therefore, incorporating the environmental administrative substitute enforcement mechanism into the implementation mechanism of the eco-environmental restoration liability clause is helpful in solving the problem of the overlap of the same plaintiff of administrative enforcement agencies and EEDCL mentioned above.

## 6. Further analysis

### 6.1. Litigation subject relation

The clause on eco-environmental restoration responsibility in the Civil Code is a public legal norm that grants two types of representative restoration rights: “government-designated agencies” and “organizations designated by law.” As the clause adopts the word “or,” it means that a choice can be made between the two types of subjects and social organizations under the framework of public law have certain special characteristics. From the perspective of traditional civil litigation theory, the litigation subject status of the parties is determined by the attribution of substantive rights and obligations, and specific subjects can only obtain the right to litigate based on “objective legality” (38). As regards environmental organizations representing the public, Article 58 of the EPL clearly stipulates the main purpose of the legal environmental protection organizations’ environmental public welfare maintenance and allows them to file EPIL, but this type of litigation is still a remedy for ecological environment damage under the private law framework. With the establishment of the object of “violation of national regulations, “the adjusted legal relationship should be transformed into an administrative one, and the designated social organization should be authorized by the state.” According to administrative law regulations, such authorities mainly involve “administrative agencies stipulated by law” and “other organizations authorized by laws and regulations.” Among them, the authorized subject can also realize some of the administrative functions through certain management or autonomous actions. A typical example in China is the neighborhood or village committee, which is guided by the street office or township government and authorized by the corresponding organizational law to perform various administrative functions. Environmental organizations belong to social groups and are characterized by having a large number of members and an obvious public nature. According to Article 58, Paragraph 2 of the Civil Procedure Law, environmental organizations have priority over the procuratorate in initiating litigation to safeguard environmental public welfare, while at the same time; the order of priority of administrative agencies is clearly defined in the Provisions Promulgated by the SPC on Hearing Cases Concerning Compensation for eco-environmental damage (Trial). Therefore, the order of proceedings between environmental organizations and administrative agencies and procuratorates needs to be adjusted along with the adjustment of administrative legal relationships.

According to the basic principles of administrative law, public authority is generally not exercised by private organizations outside the administrative agencies (39). The reason why legislators choose to entrust public affairs to social organizations is more based on the need for “representative enforcement,” that is, when the system of eco-environmental restoration has not been fully established within the framework of public law, the private law relief path of converting the costs of prevention or restoration measures into property rights losses is chosen. Currently, the obligation of administrative agencies in China is further strengthened through means such as administrative orders, supervision, and coercion, and the objective conditions for social organizations to perform restoration measures in a dominant position have changed. On the one hand, administrative agencies prioritize the implementation of eco-environmental restoration measures on behalf of others. To a certain extent, the representative performance is due to the breach of the primary obligation by the parties, and the payment of the applicable fee is the secondary obligation that the parties are responsible for (40). Therefore, the administrative agency should carry out the notice and representative restoration procedures, and the consultation procedures should focus on the compensation for the representative performance fee. Only when the administrative agency cannot complete the restoration measures can the judicial route be used to play the role of ecological environment damage relief. On the other hand, the procuratorial organs have priority in initiating litigation over social organizations. The priority of the latter organizations in safeguarding environmental public welfare has also revealed a certain degree of inadaptability in practice, because the implementation of eco-environmental restoration is purely public welfare-oriented, and social organizations cannot profit from initiating litigation, judgment enforcement, etc., and they also have to bear additional economic expenses (investigation and evidence collection costs, lawyer fees, etc.) to conduct litigation, making it difficult to advance the civil public welfare litigation procedure for the environment. In practice, there are situations where social organizations request the procuratorial organs to transfer evidence and require the defendant to bear lawyer fees after these organs have initiated litigation. Based on the function of legal supervision, the latter have more professional advantages in the process of carrying out their duties such as investigating and collecting evidence and initiating litigation (41).

It is worth noting that the adjustment of social organizations does not exclude public participation. Based on public health considerations, “those most affected who are potentially harmed by the outcome of the risk should have [the] right to speak in determining the level of risk they and their society can tolerate” (42). In other words, it is necessary to listen to the public opinions in the affected areas, regardless of the stage of ecological damage relief.

### 6.2. Comparison of judicial elements

Once environmental pollution causes group-type health damage, its consequences are serious or even irreversible. For example, if children are exposed to excessive levels of lead for a long time, it can cause mental impairment and affect them for the rest of their lives (43). It can be seen that “right relief, damage repair” cannot fully deal with environmental and health problems, and the normative concept of post-relief needs to be altered to risk prevention. At present, on the basis of the provision of responsibility for ecological and environmental restoration already established in the Civil Code, the preventive relief function should be further expanded and integrated into the consideration of elements of public health protection.

Firstly, Article 1,234 of the Civil Code should be optimized. By adding the category of “major risks,” the relief system for ecological environmental damage under the Civil Code can be given a preventive function so as to cover the consequences of environmental pollution or ecological damage to public health caused by large-scale activities (nuclear energy regulation, management of climate change, and highly toxic substances management etc.). Legal organs or social organizations can require actors to eliminate major ecological environment risks. Combined with the above-mentioned adjustment of the implementation mechanism of the eco-environmental restoration, the administrative organ should immediately require the subject to eliminate the potential risks when they violate the state regulations and there may be major environmental risks. If the latter are removed, there is no need to start the subsequent damage relief procedures.

Secondly, the criteria for determining the correlation between environmental risks and public health should be established. Because there are no objective criteria for judging ecological environmental risks and environmental public welfare, there are not many cases of preventive relief in practice. In the face of complex and diverse harm to environmental health, traditional tort damage relief can only fill in the category of individual health damage, and the impact of group health damage often cannot be supported because of its complexity. However, the ecological environment usually refers to the interests of an unspecified majority of people, and such risks cannot be reduced to specific individual rights and interests. Therefore, the correlation consideration of “public health” elements should be added in the judgment of environmental risks, including the water environment benchmark, atmospheric environment and soil environment benchmarks established based on public health, which are all important reference indicators.

Thirdly, the environmental health risk system should be used to carry out pre-litigation procedures and case reviews. Prior to the establishment of the relief system for EED in the Civil Code, Article 1 of the EPL specifically set out the objective of “safeguarding public health,” and Article 39 also provided for the environmental health risk assessment system. In 2018, the former Ministry of Environmental Protection (now the Ministry of Ecology and Environment) issued the National Working Measures on Environment and Health for Environmental Protection (Trial), which stipulated the system for the monitoring, investigation, and risk assessment of environment and health in detail, thus establishing a relatively complete system for environmental health protection. Prior to the initiation of the proceedings, the legal subject shall proceed in strict accordance with the urge procedure or consultation procedure, and make objective judgments on risks or damages by means of environmental and health monitoring, investigation and risk assessment systems. In addition, administrative organs should integrate public health into the existing environmental protection systems (i.e., for total emission control of major pollutants, environmental impact assessment system, emergency response to environmental incidents, etc.) and strengthen the examination of environmental risks through the operation of these systems so as to realize a comprehensive consideration of health factors. And the court will also judge the case in combination with the corresponding system at the case review stage, so as to decide whether to file the case rather than examine the abstract environmental interests under the broad concept of risk prevention.

### 6.3. Assumption of the use of funds

Based on the relief system of EED under the framework of civil law, its realization is mainly to request the parties to take measures to restore the ecological environment or pay compensation when they fail to do so, which can be summed up as “the principle of behavioral responsibility and the exception of economic responsibility.” Therefore, the court collected a large amount of compensation for EED, and each region gradually realized the compensation for EED and promulgated local normative documents one after another to regulate the use of funds.

The purpose of compensation for EED mainly includes four categories: first, expenses for emergency response to EED, including pollution removal, emergency monitoring, emergency treatment, and expert guidance; second, compensation for ecological and environmental restoration losses, including the cost of compensation for the loss of service functions during ecological and environmental restoration and the cost of compensation for the loss caused by permanent damage to the ecological environment; third, the cost of ecological and environmental restoration or alternative restoration, including the preparation of such restoration plans, construction, supervision, government procurement, and evaluation of restoration effects; fourth, litigation expenses, such as investigation and evidence collection, appraisal, and lawyer representation required by the organization or institution to initiate litigation. Some areas have added other uses, such as spending on environmental protection public welfare activities and rewards for individuals or organizations that have made significant contributions to ecological environment protection (see Table 2).

**Table 2:** Provisions on the use of compensation for eco-environmental damage in some areas.

Area	Specific regulations
Zhejiang, Guangxi, Ningxia, and Qinghai	Cost of cleaning up pollution; (2) EED repair costs (including alternative repair costs); (3) Reasonable expenses for investigation, appraisal and evaluation of EED compensation, and post assessment of restoration effects; and (4) Other expenses related to ecological environment damage compensation as stipulated by laws and regulations.
Guizhou	Emergency response costs for ecological environment damage; (2) Costs for cleaning or controlling pollution; (3) Cost of ecological environmental remediation or alternative restoration; (4) Necessary expenses for conducting investigations and evidence collection, appraisal and evaluation, investigation, environmental monitoring, expert consultation, lawyer representation, and litigation related to EED; and (5) Other expenses specified in laws, regulations and normative documents for the restoration of ecological environment damage.
Jiangsu	Expenditures related to clearing or controlling the pollution (including emergency response); (2) Expenditure for repairing EED; (3) Expenses related to appraisal and evaluation (including investigation, appraisal, inspection, monitoring, expert consultation), preparation of restoration plans and assessment of restoration effects, lawyer agency, and litigation in ecological environment damage compensation litigation initiated by the department or institution designated by the compensation right holder; (4) Other expenses related to the restoration of ecological environment damage; and (5)The compensation funds paid for EPIL shall be used according to the prescribed purposes if the effective legal documents specify the purpose.
Shenzhen	Expenditures for alternative restoration of EED, including the preparation, implementation, and evaluation of restoration plans; (2) Expenses related to investigation and evidence collection, appraisal and evaluation, litigation fees, lawyer agency, etc. required for initiating environmental civil public interest litigation;(3) Relevant expenses with clear purposes specified in effective legal documents;(4) Reward expenditures for organizations and individuals who have made significant contributions to the protection of the ecological environment; (5) Relevant expenses incurred during the emergency response phase; and (6) Expenditure on public welfare activities for ecological and environmental protection.
Hunan	Cost for pollution removal, eco-environmental restoration, and effect assessment of ecological environmental remediation; (2) Compensation fees for the loss of service functions during the restoration of ecological environment damage; (3) Compensation fees for losses caused by permanent damage to ecological environment functions; (4) Cost of investigation, identification, and evaluation of ecological environment damage; (5) Litigation costs for EED compensation (including public interest litigation); and (6) Reasonable expenses incurred to prevent the occurrence and expansion of damage.
Anhui, Hebei	Cost of cleaning or controlling pollution; (2) Cost of ecological environmental remediation or cost of alternative restoration; (3) Compensation for the loss of service functions during the period from ecological environment damage to restoration; (4) Compensation for losses caused by permanent damage to ecological and environmental functions; (5) Reasonable expenses for the preparation of ecological environment damage remediation plans and post assessment of ecological environment damage remediation; (6) Necessary fees for investigation and evidence collection, expert consultation, environmental monitoring, appraisal, survey, audit, evaluation, acceptance, lawyer agency, etc.; and (7) Other related expenses required by laws and regulations for the restoration of ecological environment damage.
Xinjiang, Yunnan	Compensation fees for losses caused by permanent damage to ecological and environmental functions; (2) Compensation fees for the loss of service functions during the period from ecological environment damage to restoration; (3) Cost of cleaning or controlling pollution; (4) The cost of reasonable emergency response measures taken to prevent the occurrence and expansion of ecological environment damage; (5) EED repair costs or alternative repair costs; (6) Expenses for investigation and evidence collection, exploration and appraisal, environmental monitoring, expert consultation and evaluation, preparation of restoration plans and assessment of restoration effects, hiring lawyers and litigation organized by the designated department of the compensation rights holder; and (7) Other relevant expenses stipulated by laws and regulations.

Obviously, the compensation for EED has the characteristics of public welfare and special purpose. It should follow the principle of “special funds for special purpose” and be used for the specific purpose of EED restoration that is, the compensation for EED should be related to the restoration measures after the ecological environment has been damaged, which must include the scope and degree of damage caused by the ecological environment destruction. Based on the correlation between public health and EED, the relief of EED aims to protect the former from being infringed. Therefore, public health protection should be included in the scope of fund use. Specifically, it can be included in the “cost of service function loss during ecological and environmental restoration” as stipulated in Article 1,235 of the Civil Code. If the damage to the ecological environment affects the health and safety of nearby residents and other subjects, the compensation need covers these.

The specific use method comprises two approaches. The first is based on the fact that the main body in the application for compensation for ecological environmental damage is the environmental administrative department, which includes the department of natural resources and that of the ecological environment, which should increase the protection measures for public health when carrying out restoration with compensation, and establish a comprehensive restoration plan. As for the supplementary subject of compensation application, environmental protection organizations can also apply for compensation to carry out restoration measures on the basis of full investigation of the damaged ecological environment. In the second approach, the surrounding residents can apply for compensation after the health damage has been confirmed by the appraisal, and this part of the compensation standard can be basically restored to the original living conditions. In this case, residents should be excluded from claiming compensation through environmental tort litigation, but should apply when the liable subject is unknown or unable to pay.

## 7. Conclusion

Protecting public health is the legislative purpose of China’s EPL, and strengthening compensation for environmental health damage conforms to the purpose of China’s Basic Law on environmental protection. In situations where the right to environmental health has not yet been explicitly defined as a right by positive law, there is an urgent need to make full use of existing institutional resources. Since the promulgation and implementation of the Civil Code on May 28, 2020, China’s EED relief system has taken initial shape and is capable of preventing damage to, and relieving, public health. However, only relying on traditional tort rules to carry out ecological environmental damage relief cannot effectively cover the scope of public health. From the perspective of public law, it is helpful to identify various types of subjects and integrate existing relief mechanisms. Further, in order to strengthen the protection of public health, it is necessary to adjust the order of the replacement restoration subjects of ecological environmental damage, integrate the factors of public health risk into the relief procedures, and increase the use of ecological environmental damage compensation for public health protection. Of course, the protection of public health is not limited to this. In the future, it will also be necessary to continuously enrich the content of the management system related to public health risks and damages, and improve the connection mechanism between administrative regulation and judicial relief so as to give play to the positive role of various subjects in protecting public health.

## Data availability statement

The original contributions presented in the study are included in the article/supplementary material; further inquiries can be directed to the corresponding author.

## Author contributions

HL: original draft preparation, supervision, and funding acquisition. BL: investigation, visualization, and supervision. All authors contributed to the article and approved the submitted version.

## Funding

This research received funding from the National Social Science Fund Youth Project “Legal Application of Green Provisions in the Implementation of the Civil Code” (21CFX046).

## Conflict of interest

The authors declare that the research was conducted in the absence of any commercial or financial relationships that could be construed as a potential conflict of interest.

## Publisher’s note

All claims expressed in this article are solely those of the authors and do not necessarily represent those of their affiliated organizations, or those of the publisher, the editors and the reviewers. Any product that may be evaluated in this article, or claim that may be made by its manufacturer, is not guaranteed or endorsed by the publisher.

## Footnotes

^1^Since October 2008, there has been a series of heavy metal pollution incidents in Hechi City, Guangxi Zhuang Autonomous Region, which have seriously damaged public health. On January 15, 2012, the normal level of cadmium was seriously exceeded in Longjiang River in Hechi City, which not only affected production and life along the river, but also seriously threatened the drinking water safety of Liuzhou residents, resulting in an extremely bad social impact. See the official website of the Ministry of Ecology and Environment: Notice on Suspension of Environmental Impact Assessment Approval for Construction Projects in Hechi City, Guangxi Zhuang Autonomous Region. Available at: https://www.mee.gov.cn/gkml/hbb/bgth/201202/t20120213_223415.htm See the website of the People’s Government of the People’s Republic of China: "The Pain of Unscientific Development in the Occurrence of Cadmium Pollution in Longjiang, Guangxi". Available at: https://www.gov.cn/govweb/jrzg/2012-01/31/content_2055316.htm (Accessed May 25, 2023).

^2^Refer to the Civil Ruling No. 1057 of Hunan High People’s Court (2016).

^3^The Xiangshui "March 21" explosion in Jiangsu Province, occurred on March 21, 2019. The direct cause of the accident was spontaneous combustion caused by continuous heating of nitrification waste illegally stored for a long time in the old solid waste storage of Tianjiayi Company, which resulted in combustion and explosion. 78 people were killed and 76 seriously injured. Available at: https://baijiahao.baidu.com/s?id=1684757212494315157&wfr=spider&for=pc (Accessed May 26, 2023).

^4^In 2007, the National Action Plan on Environment and Health (2007–2015), jointly formulated by 18 departments including the Ministry of Health, SEPA, and the National Development and Reform Commission, used various expressions such as "compensation for environmental pollution damage," "health damage caused by environmental pollution," and so on. In 2015 and 2017, the General Offices of the CPC Central Committee and the State Council jointly issued the Pilot Plan for the reform of the compensation system for Ecological and Environmental Damage, which clearly stated that "local governments should be encouraged to carry out exploratory research and practice on compensation for environmental health damage." In 2017, the former Ministry of Environmental Protection issued the National Environmental Protection 13th Five-Year Plan on Environment and Health Work, proposing to study the "environmental pollution health damage assessment technology, methods and compensation mechanism," again using the term "environmental pollution health damage compensation." In 2018, the Working Measures on Environment and Health issued by the former Ministry of Environmental Protection defined environmental health risk for the first time as: "[it] refers to the possibility of environmental pollution (biological, chemical and physical) causing adverse effects on public health, and the qualitative or quantitative estimation of this possibility is called environmental health risk assessment."

^5^As for the legal nature of technological modification deduction, there are some viewpoints: "the way of liability," "the way of judgment execution," "the way of loss compensation," and "the way of liability relief."

^6^Refer to Civil Judgment No. 2299 of Kunming Intermediate People’s Court (2017).

^7^Refer to the Civil Judgment No. 114 of the Intermediate People’s Court of Pingxiang City, Jiangxi Province (2018).

^8^Refer to the Administrative Judgment No.0981-0071 of Fu’an City People’s Court, Fujian Province (2018).
